# Fluorescein Angiographic Findings of Peripheral Retinal Vasculature after Intravitreal Conbercept versus Ranibizumab for Retinopathy of Prematurity

**DOI:** 10.1155/2019/3935945

**Published:** 2019-12-31

**Authors:** Enzhong Jin, Hong Yin, Yufei Gui, Juan Chen, Jian Zhang, Jianhong Liang, Xiao-xin Li, Mingwei Zhao

**Affiliations:** ^1^Department of Ophthalmology, Ophthalmology & Optometry Center, Peking University People's Hospital, Beijing, China; ^2^Beijing Key Laboratory of Diagnosis and Therapy of Retinal and Choroid Diseases, Beijing 100044, China; ^3^Department of Ophthalmology, Beijing Jingmei Group General Hospital, Beijing, China

## Abstract

**Purpose:**

To evaluate angiographic findings of peripheral retina vasculature in retinopathy of prematurity (ROP) neonates who received intravitreal conbercept (IVC) or ranibizumab (IVR).

**Methods:**

Fluorescein angiography (FA) findings were retrospectively evaluated for ROP neonates who received IVC or IVR. Outcome measures included peripheral avascular zone, vascular leakage, vascular blunting, vascular loops, vascular dilatation, arteriovenous shunt, and capillary dropout.

**Results:**

Fifty-four eyes (28 patients) with ROP were included. Twenty-nine eyes (15 patients) received IVC, and 25 eyes (13 patients) received IVR. For infants of the IVC group, the mean gestational age, birth weight, and postmenstrual age (PMA) at the initial treatment were 28.96 ± 2.36 weeks, 1168.8 ± 344.5 g, and 41.22 ± 4.39 weeks, respectively. For the IVR group, they were 28.83 ± 2.34 weeks, 1255.0 ± 356.9 g, and 39.42 ± 2.77 weeks, respectively (*P*=0.817, 0.522, and 0.075). For the IVC group, FA performed at 71.29–115.43 weeks PMA showed 96.55% of eyes had avascular zone; vascular leakage was found in 24.14% eyes; vascular blunting, vascular dilation, vascular loops, arteriovenous shunt, and capillary dropout were found in 96.55%, 72.41%, 79.31%, 48.28%, and 68.97% eyes, respectively. For the IVR group, FA performed at 65.57–133.71 weeks PMA showed 92.0% of eyes had avascular zone; vascular leakage was found in 40.0% eyes; vascular blunting, dilatation, loops, arteriovenous shunt, and capillary dropout were found in 100%, 60.0%, 64.0%, 36.0%, and 68.0% eyes, respectively.

**Conclusion:**

No significant difference can be observed between the IVC group and IVR group for peripheral vascular structure anomalies with FA evidence. FA studies should be considered to assess the status of the peripheral retinal vasculature to determine therapeutic outcomes and potential functional outcomes.

## 1. Introduction

Retinopathy of prematurity (ROP) is a vascular disorder of immature retina developed in premature infants. Although several treatment strategies such as laser and anti-VEGF agents used for ROP have improved the clinical outcomes, ROP remains one of the leading causes of childhood blindness [1, 2].

Vascular endothelial growth factor (VEGF) dysregulation associated with hypoxia plays an important role in the pathogenesis of ROP and induced abnormal vasculogenesis and neovascularization [3–5]. For the past two decades, laser photocoagulation which is used to destruct the avascular retina was recommended for the treatment of ROP [6]. But the side effect of this treatment including visual field loss caused by permanent ablation of the hypoxic retina and subsequent high myopia should not be ignored [7, 8]. The off-label intravitreal injection of anti-VEGF agents such as conbercept (KH902; Chengdu Kanghong Biotech Co, Ltd, Sichuan, China), bevacizumab (Genentech, Inc, South San Francisco, CA), ranibizumab (Genentech, Inc and Novartis International AG, Basel, Switzerland), and aflibercept (Regeneron Pharmaceuticals Inc) for ROP treatment has been reported in recent years and shown to be efficacious [9–13]. For most ROP infants, anti-VEGF drugs could effectively improve the structural outcomes in no shorter than 6-month follow-up period although some recurrences may occur in 2.8%–35.7% of them [9–13]. But the long-run vascular development after intravitreal injections of anti-VEGF agents still requires further study.

The efficacious of anti-VEGF agents have been certified in several studies and the short-term safety was also described, while the peripheral vascular abnormalities of ROP treated with bevacizumab and ranibizumab had also been reported in recent years [14–17]. The most common vascular abnormalities include incomplete vascularization [14, 15], vascular leakage [16, 17], vascular dilation, vascular blunting [16], and capillary dropout [16].

Fluorescein angiography (FA), as an imaging modality better illustrating the vascular structure, could provide more useful information for the observation of premature retina after treatment. A number of investigators have described FA outcomes in ROP after treatment of laser and intravitreal injection of ranibizumab (IVR) or bevacizumab (IVB) [16–18]. But until now, no study has reported the FA outcomes of ROP for infants treated with intravitreal injection of conbercept (IVC). Conbercept is a recombinant fusion protein composed of the second Ig domain of VEGFR1 and the third and fourth Ig domains of VEGFR2 to the constant region (Fc) of human IgG1 and has been approved by the China Food and Drug Administration (CFDA) for intraocular application [10]. Though both conbercept and ranibizumab are efficacious for ROP treatment, there are structural and mechanism differences between them. Up to now, there is no comparison report of retinal vascular structure outcomes described by FA for ROP patients treated with conbercept and ranibizumab.

The current study aims to compare the retinal vascular outcomes identified with FA examination after monotherapy of IVC or IVR in patients requiring treatment. The structural outcomes and injection frequency among the patients treated with conbercept or ranibizumab are also discussed. To the best of our knowledge, it is the first time evaluating peripheral vascular changes on FA for ROP infants treated with conbercept.

## 2. Methods

This retrospective, nonrandomized, single-center, interventional study was approved by the Ethical Review Committee of Peking University People's Hospital (Beijing, China), which was conducted in accordance with the Declaration of Helsinki. Written informed consent was obtained from the parents of each infant before receiving the intravitreal injection of anti-VEGF agents.

The medical records of infants who underwent conbercept or ranibizumab treatment and received FA exams after treatment were conducted. All of them were treated in Children's Eye Center of Peking University affiliated People's Hospital in Beijing, China, between September 2014 and April 2018. For all enrolled infants, the RetCam (Clarity Medical Systems, Inc, Pleasanton, CA) fundus color photographs and fluorescein angiograms were reviewed.

All included children were Zone II Stage 2/3 ROP children with plus disease or AP-ROP and treated with IVC or IVR. Bevacizumab was not applied in this study. The included eyes of both groups fulfilled the criteria of treatment according to the guidelines of the Early Treatment for Retinopathy of Prematurity Cooperative Group [19]. The eyes without regression of plus disease or ridge with one injection had a repeat injection of conbercept or ranibizumab during the follow-up period, as well as those eyes with a recurrence of ROP after regression. The recurrence of ROP was defined as the recurrence of ridge and plus disease after the regression of them. The injection dose of both the IVC and IVR group was 0.25 mg/0.025 ml, which is half of adult dose. The injection procedure was performed by the same retina specialist (H. Y.) according to our preferred technique as previously described [10]. The clinical data and fluorescein angiograms were collected during routine care of the children. Exclusion criteria of this study included any previous cryotherapy, laser photocoagulation, or surgical procedures before receiving anti-VEGF treatment and presence of retinal detachment or any potential associated ocular or systemic disorders that may confound the outcomes of the study. Infants treated with more than one kind of anti-VEGF agent were also excluded.

Fundus fluorescein angiographies and photographs were obtained under general anaesthesia for all infants included. Each infant was scheduled for FA examination by the same skilled ophthalmologist (H. Y) once the infants reached 60 weeks postmenstrual age (PMA), and the exact time depended on the accessibility of the follow-up of each infant. A 10% solution of fluorescein was injected intravenously with a dose of 0.1 ml/kg, followed by an isotonic saline flush. The FA exam lasted about 5 minutes for each infant in our center. The primary outcomes studied were FA photogram data collected including the peripheral avascular zone, vascular fluorescein leakage, vascular blunting, vascular loops, vascular dilatation, arteriovenous shunt, and capillary dropout [20]. The avascular zone in the present study was defined as a distance of greater than 2 DD from the ora to the vascular terminus. The times of the initial IVR or IVC treatment were also recorded.

Spreadsheet and statistical software (Microsoft Excel and SPSS 19.0; IBM, Armonk, NY) were used for statistical analyses. The independent sample *t*-test and chi-square test were used for the data comparison of two groups, and a *P* value less than 0.05 was considered as statistically significant. No other special test or analysis was used in this study.

## 3. Results

Twenty-nine eyes of 15 infants in the IVC group and 25 eyes of 13 infants in the IVR group that developed Zone II Stage 2/3 ROP with plus disease or AP-ROP were finally included. For patients needed binocular treatment or retreatment, both eyes received the same anti-VEGF agent. The demographic data of both group infants are shown in Table 1.

All infants underwent FA examinations observed in the present study were included as consecutive case series in both groups. FA examinations were performed under general anaesthesia at a mean time of 88.66 ± 10.88 weeks (range, 71.29–115.43) and 98.02 ± 25.28 weeks (range, 65.57–133.71) PMA for the IVC and IVR group. The FA characteristics data were extracted from the fluorescein angiographies of the last exam for all enrolled infants and are shown in Table 2.

### 3.1. Intravitreal Injection of the Conbercept Group

Twenty-nine eyes of 15 infants with a mean gestational age of 28.96 ± 2.36 weeks (range, 26.14–33.43) received primary IVC treatment. The mean birth weight was 1168.8 ± 344.5 g (range, 850–2100), and mean age at initial conbercept injection was 41.22 ± 4.39 weeks (range, 36.29–50.14) PMA. Of the 29 eyes, 27 had Zone II Stage 3 ROP with plus disease, whereas the other 2 eyes showed AP-ROP. The included eyes received 1.28 ± 0.45 injections during the follow-up period while 8 (27.59%) of them received a second injection.

The FAs were examined ranging from 71.29 weeks to 115.43 weeks PMA for the IVC group, and the peripheral retinal vasculature was evaluated. In the IVC group, vascular fluorescein leakage was observed in 24.1% of eyes while 96.6% of eyes had avascular zones. Vascular blunting, vascular loops, vascular dilatation, arteriovenous shunt, and capillary dropout were observed in 96.55%, 79.3%, 72.4%, 48.3%, and 69.0% of eyes.

**Figure 1:**
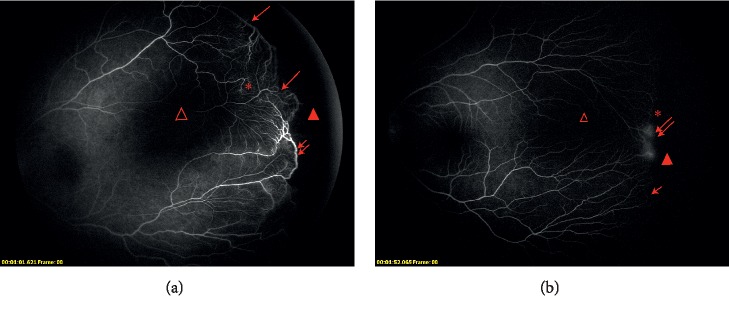
Fluorescein angiographic images of infants receive IVC therapy. (a) Fluorescein angiographic images of an infant (no. 10 in the IVC group) born at 34 weeks' gestational age who had zone II stage 3 ROP with plus disease. Primary intravitreal injection of conbercept was performed at 47.57 weeks. Fluorescein angiography at 92.14 weeks PMA exhibits persistent avascular zone (closed arrowhead), vascular dilation (double arrow), arteriovenous shunt (arrow), vascular loops (asterisk), and capillary dropout (open arrowhead). (b) Fluorescein angiographic images of an infant (no. 13 in IVC group) born at 29.43 weeks' gestational age who had AP-ROP with plus disease. Primary intravitreal injection of conbercept was performed at 36.43 weeks. Fluorescein angiography at 93.57 weeks PMA exhibits persistent avascular zone (closed arrowhead), vascular leakage (double arrow), arteriovenous shunt (arrow), vascular blunting (asterisk), and capillary dropout (open arrowhead).

The fluorescein angiographic images of an infant received IVC therapy are shown in Figure 1.

### 3.2. Intravitreal Injection of the Ranibizumab Group

Twenty-five eyes of 13 infants with a mean gestational age of 28.76 ± 2.25 weeks (range, 25.43–33.43) received primary IVR treatment. The mean birth weight was 1255.0 ± 356.9 g (range, 760–1950), and mean age at initial ranibizumab injection was 39.42 ± 2.77 weeks (range, 36.00–44.86) PMA. Among the 25 eyes, 19 of them had Zone II Stage 3 ROP with plus disease, whereas the other 6 eyes showed AP-ROP. The included eyes received 1.56 ± 0.65 injections during the follow-up period while 12 (48%) of them received at least two injections.

The FAs were examined ranging from 65.57 weeks to 133.71 weeks PMA for the IVR group. In the IVR group, vascular fluorescein leakage was evident on FA in 40.0% of eyes while 92.0% of eyes had avascular zone. Vascular blunting, vascular loops, vascular dilatation, arteriovenous shunt, and capillary dropout were observed in 100%, 64.0%, 60.0%, 36.0%, and 68.0% of eyes.

**Figure 2:**
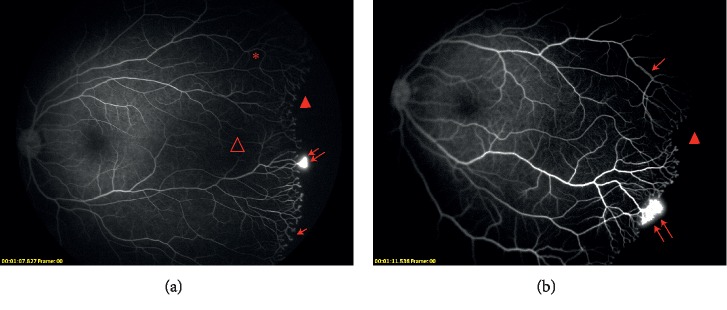
Fluorescein angiographic images of infants receive IVR therapy. (a) Fluorescein angiographic images of an infant (no. 1 in IVR group) born at 28.29 weeks' gestational age who had zone ii stage 3 ROP with plus disease. Primary intravitreal injection of ranibizumab was performed at 38.86 weeks. Fluorescein angiography at 66.29 weeks PMA exhibits persistent avascular zone (closed arrowhead), vascular leakage (double arrow), vascular blunting (arrow), vascular loops (asterisk), and capillary dropout (open arrowhead). (b) Fluorescein angiographic images of an infant (No. 7 in IVR group) born at 27.86 weeks' gestational age who had Zone II Stage 3 ROP with plus disease. Primary intravitreal injection of ranibizumab was performed at 39.00 weeks. Fluorescein angiography at 70.86 weeks PMA exhibits persistent avascular zone (closed arrowhead), vascular leakage (double arrow) and vascular dilation (arrow).

The fluorescein angiographic images of an infant received IVR therapy are shown in Figure 2.

## 4. Discussion

The result of our present study shows the persistent vascular structural abnormalities in eyes with ROP up to 133 weeks PMA after IVR and 115 weeks PMA after IVC. This FA study mirrors previous reports of peripheral retinal vascular abnormalities [16]. To the best of our knowledge, the current study is the first case control series to show the vascular changes on FA after intravitreal conbercept therapy for ROP and compare the vascular abnormalities with infants treated with ranibizumab.

In the past decade, laser photocoagulation was performed as the first-line treatment for ROP and reduced the unfavorable visual acuity outcomes by ablating peripheral avascular retina [19]. In recent years, the application of anti-VEGF agents has indeed improved the structural outcomes of ROP. But still, some cases need further laser treatment or even surgery [10, 13, 21]. The potential vascular abnormalities continued after anti-VEGF treatment may have persistent implications for the retinal structure and visual function. Even experienced retinal specialists with favorable examination settings could not precisely observe the peripheral vascular anomalies by binocular indirect ophthalmoscopy, especially for the vascular leakage that can only be identified by FA exam. Undoubtedly, FA can better identify the vascular changes for the follow-up of ROP and help to perform the necessary laser treatment. Since 2006, the safety of FA application in neonates has been described [22]. Several studies then reported the diagnosis, treatment, and follow-up with FA in ROP management [17, 20, 23, 24]. The present study focuses on the angiographic findings after primary treatment of IVC or IVR and tries to better describe the vasculature outcomes of ROP after anti-VEGF treatment.

In this study, the FA exams were performed at a similar time frame for the IVC group and IVR group, which were similar or slightly later than that of the previous studies. As was concerned in Harper et al.'s study, FAs were performed ranging from 44 weeks to 150 weeks PMA [16]. And in Lepore et al.'s study, the FA findings were collected 9 months after treatment [17]. All enrolled eyes of our present study underwent FA by 60 weeks PMA when possible, because of the previous report of persistent vascular arrest occurred at this point after primary anti-VEGF treatment for ROP [15].

FA exams allow better observation of vascular-avascular junction and help in estimating the avascular retina [17]. In a previous study [25], 33 eyes from 31 normal children were estimated using the RetCam FA for avascular retina. Since none of the included eyes has a distance greater than 1.5 disk diameters (DD) from vascularised retinal margin to the ora serrata up to 13 years of age, the authors conclude 2 DD to the ora serrata as abnormal avascular terminate. The avascular zone in the present study is also defined as a distance of greater than 2 DD from the ora to the vascular termini. In our study, 96.55% (28/29) and 92.0% (23/25) of enrolled eyes had avascular zone until the last FA exam in the IVC and IVR group. The majority of eyes do not reach vascularization within 2 DD of the ora serrata. Not all the eyes with avascular retina require treatment except those with persistent vascular leakage, which can be defined by FA.

In a recent study evaluating the FA findings of eyes primarily treated with ranibizumab, peripheral avascularity-associated vascular abnormalities like loops, blunting, dilatation, and vascular leakage were identified [16]. They reported a total of 40% persistent vascular leakage, 90% vascular dilation or blunting, and 93.4% capillary dropout in all ranibizumab-treated eyes. For the IVR group of the present study, similar outcomes with the reported results are found. Forty percent of the included eyes are identified to have vascular leakage while all enrolled eyes present vascular blunting. Vascular loops/dilation is found in 64%/60.0% eyes, which is fewer than that reported in the published study [16]. Besides, only 68.0% eyes present capillary dropout, which is much fewer than that of previous study. For the IVC group, vascular leakage is observed in 24.1% of eyes, which seems fewer than that reported in previous studies and the IVR group in the present study but with no statistically significance (*P*=0.211). Vascular blunting and vascular loops/dilation are also similar to that reported in previous study and our IVR group [16]. Just like the IVR group, capillary dropout is observed in only 69.0% eyes, which is much fewer than that of the previous study [16]. Arteriovenous shunt as described in Lepore's study is also identified in our study [17]. In the IVC group, the arteriovenous shunt is found in 48.3% eyes while 36.0% eyes of the IVR group also present that. The junctions between vessels always locate between vascular and avascular retina and could be the starting line of avascular zone. Since the arteriovenous shunt makes the arterial and venous blood flow communication, we summarize that vessels in most eyes presenting such a manifestation would not develop toward the periphery any more. But this hypothesis still requires further observation with longer duration of follow-up. Although there are structural differences between conbercept and ranibizumab, no difference was found in our previous study for ROP treatment [10]. Meanwhile, no significant difference can be observed between the IVC group and IVR group for peripheral vascular structure anomalies in our study (Table 3). As two different anti-VEGF agents, both conbercept and ranibizumab are used to decrease the VEGF level of ROP eyes, which is thought to affect the vascular development of premature infants. According to our previous study [10] and the present study, we hypothesis that similar efficacy for ROP treatment and effect on vascular development may be defined for conbercept and ranibizumab. But stronger evidence still requires a better design head-to-head RCT research.

Both timely treatment and suitable follow-up are required for the administration of ROP. In our study, all infants received primary anti-VEGF treatments and underwent FA exams during the follow-up period longer than one year. Most eyes regressed with one injection and showed good retina vascular development. The effectiveness of anti-VEGF drugs has been verified in several studies [10, 12, 13] while the anomalies after treatment are also reported in recent years. Whether persistent peripheral vascular abnormalities are associated with the time or frequency of anti-VEGF treatment still require further studies.

In the present study, the IVR group receives higher injection frequency than that of the IVC group in our study but with no statistical significance. The relatively higher frequency seems to be associated with the relatively higher AR-ROP-included rate in the IVR group (3/13) than in the IVC group (1/15) (*P*=0.486). Nearly all AP-ROP eyes (5/6, 83.33%) in the IVR group receive a second injection during the follow-up period. But on the other hand, the non-AP-ROP patients requiring more than 1 injection in these two groups are 7/25 and 7/29, with no significant difference. Since the included sample is not large enough, further research with larger scale is still needed.

There are several notable limitations in our present study that must be mentioned. Firstly, it is a retrospective study and not randomized. Only the eyes receive anti-VEGF treatments were collected, but no matched laser-treated group is designed. Secondly, the numbers of the included infants in both groups are relatively small, leading to lower strength of convincing. Thirdly, the postmenstrual age at time of FA was variable among infants, which cannot be strictly unified in a retrospective study. Undoubtedly, prospective studies with a larger sample size are required and more cases remain to be collected.

In summary, the present study contributes to the fluorescein angiographic findings of ROP treated with different anti-VEGF agents, which improves the accuracy of peripheral retina vascular observation. The first-time description of FA findings for ROP infants treated with conbercept provides more data for its clinical application. Besides, FA has been reported in the diagnosis and management of ROP in recent years, plays an important role, and also shows its advantage in the observation and judgement of retreatment for ROP compared to fundus photographs. Larger sample studies for utility of fluorescein angiography in ROP are required to elucidate better application of FA in ROP.

## Figures and Tables

**Table 1:** Baseline characteristics of retinopathy of prematurity infants receiving intravitreal conbercept or ranibizumab.

	Intravitreal conbercept	Intravitreal ranibizumab	*P*
No. of eyes (infants)	29 (15)	25 (13)	—
Mean GA (weeks)	28.96 ± 2.36 (26.14–33.43)	28.76 ± 2.25 (25.43–33.43)	0.817
Mean BW (g)	1168.8 ± 344.5 (850–2100)	1255.0 ± 356.9 (760–1950)	0.522
Male sex (%)	60%	61.54%	0.934
Mean PMA at first treatment	41.22 ± 4.39 (36.29–50.14)	39.42 ± 2.77 (36.00–44.86)	0.075
Mean injection times	1.28 ± 0.45 (1–2)	1.56 ± 0.65 (1–3)	0.074
Mean PMA at FA (weeks)	88.66 ± 10.88 (71.29–115.43)	98.02 ± 25.28 (65.57–133.71)	0.251

GA, gestational age; BW, birth weight; PMA, postmenstrual age; FA: fluorescein angiography. Data are mean ± SD(range).

**Table 2:** Fluorescein angiographic findings after primary intravitreal conbercept or ranibizumab in neonates with ROP.

Group	Patient no.	Diagnosis	GA, weeks	BW, g	PMA at FA, weeks	Eyes	Injection times	Fluorescein angiography findings						
								Avascular zone	Leakage	Blunting	Dilation	Loops	Arteriovenous shunt	Capillary dropout
C	1	Zone II Stage 3+	28	1080	94.14	OD	2	Y	Y	Y	Y	Y	N	Y
						OS	2	Y	Y	Y	Y	Y	Y	N
C	2	Zone II Stage 3+	26.14	937	98.86	OD	2	Y	N	Y	Y	Y	Y	Y
						OS	2	Y	Y	Y	Y	Y	N	Y
C	3	Zone II Stage 3+	28	1175	85.86	OD	1	N	N	Y	N	N	N	N
						OS	1	Y	N	Y	Y	Y	Y	Y
C	4	Zone II Stage 3+	28	1360	90.86	OD	1	Y	N	Y	Y	N	Y	Y
						OS	1	Y	N	Y	Y	N	Y	Y
C	5	Zone II Stage 3+	28.43	850	82.57	OD	2	Y	N	Y	Y	Y	N	Y
						OS	2	Y	N	Y	Y	Y	N	Y
C	6	Zone II Stage 3+	29.43	850	84.71	OD	1	Y	N	N	N	N	N	Y
						OS	1	Y	N	Y	Y	Y	N	Y
C	7	Zone II Stage 3+	26.86	920	76.86	OD	2	Y	N	Y	N	Y	N	Y
						OS	2	Y	N	Y	Y	Y	Y	N
C	8	Zone II Stage 3+	29.29	1550	80.57	OD	1	Y	N	Y	N	N	N	Y
						OS	1	Y	N	Y	N	Y	Y	Y
C	9	Zone II Stage 3+	26.43	1010	71.29	OD	1	Y	N	Y	Y	Y	N	N
						OS	1	Y	N	Y	Y	Y	N	N
C	10	Zone II Stage 3+	34	860	92.14	OD	1	Y	N	Y	Y	N	N	Y
						OS	1	Y	Y	Y	Y	Y	Y	Y
C	11	Zone II Stage 3+	33.43	2100	115.43	OD	1	Y	Y	Y	Y	Y	N	N
						OS	1	Y	N	Y	Y	Y	Y	Y
C	12	Zone II Stage 3+	30.29	1350	95.29	OD	1	Y	N	Y	Y	Y	N	Y
						OS	1	Y	N	Y	N	Y	N	Y
C	13	AP-ROP	29.43	1200	93.57	OD	1	Y	N	Y	N	Y	Y	Y
						OS	1	Y	Y	Y	N	Y	Y	Y
C	14	Zone II Stage 3+	26.43	890	78	OD	1	Y	N	Y	Y	Y	Y	N
						OS	1	Y	Y	Y	Y	Y	Y	N
C	15	Zone II Stage 3+	30.29	1400	95.29	OD	1	Y	N	Y	Y	Y	Y	N
R	1	Zone II Stage 3+	28.29	1100	66.29	OD	2	Y	N	Y	Y	Y	Y	N
						OS	2	Y	Y	Y	Y	Y	N	Y
R	2	Zone II Stage 3+	27.71	1150	65.57	OD	1	Y	Y	Y	Y	Y	Y	Y
						OS	1	Y	Y	Y	Y	Y	N	Y
R	3	Zone II Stage 3+	27.43	1100	71.57	OD	1	Y	Y	Y	Y	Y	Y	N
						OS	1	Y	Y	Y	N	Y	N	Y
R	4	Zone II Stage 3+	28.43	1190	110.86	OD	1	Y	N	Y	Y	N	N	Y
						OS	1	Y	N	Y	Y	N	N	Y
R	5	Zone II Stage 3+	26.29	1000	71.57	OD	1	Y	N	Y	N	Y	N	N
						OS	2	Y	Y	Y	Y	N	N	Y
R	6	Zone II Stage 2+	28.86	1250	116.71	OD	1	Y	N	Y	N	N	N	N
						OS	1	Y	N	Y	Y	Y	Y	N
R	7	Zone II Stage 3+	27.86	1070	70.86	OD	3	Y	N	Y	Y	Y	N	Y
						OS	3	Y	Y	Y	Y	Y	Y	N
R	8	Zone II Stage 3+	25.43	900	133.71	OD	2	Y	N	Y	Y	N	Y	N
						OS	2	Y	N	Y	N	N	N	Y
R	9	Zone II Stage 3+	27.86	760	120.29	OD	1	N	N	Y	N	N	N	N
		Zone II Stage 2+				OS	1	Y	N	Y	N	N	N	Y
R	10	AP-ROP	32.57	1950	110	OD	1	Y	N	Y	Y	Y	Y	Y
						OS	2	Y	Y	Y	N	Y	N	N
R	11	AP-ROP	29.57	1500	122.86	OD	2	Y	N	Y	Y	Y	Y	Y
						OS	2	Y	N	Y	N	Y	Y	Y
R	12	AP-ROP	33.43	1900	95.43	OD	2	N	Y	Y	N	Y	N	Y
						OS	2	Y	N	Y	N	N	N	Y
R	13	Zone II Stage2+	30.14	1445	118.57	OD	1	Y	Y	Y	Y	Y	N	Y

Group C, treated with conbercept; Group R, treated with ranibizumab; GA, gestational age; BW, birth weight; PMA, postmenstrual age; FA, fluorescein angiography; AP-ROP, aggressive posterior retinopathy of prematurity.

**Table 3:** Number of eyes (%) with peripheral retinal vasculature abnormalities for ROP infants receiving intravitreal conbercept or ranibizumab.

	Intravitreal conbercept	Intravitreal ranibizumab	*P*
Avascular zone, *N* (%)	28 (96.55%)	23 (92.00%)	0.591
Leakage, *N* (%)	7 (24.14%)	10 (40.00%)	0.689
Blunting, *N* (%)	28 (96.55%)	25 (100.00%)	1.0
Dilation, *N* (%)	21 (72.41%)	15 (60.00%)	0.335
Loops, *N* (%)	23 (79.31%)	16 (64.00%)	0.210
Arteriovenous shunt, *N* (%)	14 (48.28%)	9 (36.00%)	0.363
Capillary dropout, *N* (%)	19 (68.97%)	17 (68.00%)	0.847

## Data Availability

The data used to support the findings of this study are available from the corresponding author upon request.
